# Identification of DNA methylation biomarkers for risk of liver metastasis in early-stage colorectal cancer

**DOI:** 10.1186/s13148-021-01108-3

**Published:** 2021-06-09

**Authors:** Weihua Li, Lei Guo, Wanxiangfu Tang, Yutong Ma, Xiaonan Wang, Yang Shao, Hong Zhao, Jianming Ying

**Affiliations:** 1 Department of Pathology, National Cancer Center/National Clinical Research Center for Cancer/Cancer Hospital, Chinese Academy of Medical Sciences and Peking Union Medical College, No. 17 Panjiayuan Nanli, Beijing, 100021 China; 2 Nanjing Geneseeq Technology Inc., Nanjing, Jiangsu China; 3 Department of Hepatobiliary Surgery, National Cancer Center/National Clinical Research Center for Cancer/Cancer Hospital, Chinese Academy of Medical Sciences and Peking Union Medical College, Beijing, 100021 China

**Keywords:** Colorectal cancer, Liver metastasis, DMRs, DNA methylation, Biomarker

## Abstract

**Background:**

Liver metastases can occur even in CRC patients who underwent curative surgery. While evidence suggested that adjuvant chemotherapy can help to reduce the occurrence of liver metastases for certain patients, it is not a recommended routine as the side effects outweigh the potential benefits, especially in Stage II CRC patients. This study aims to construct a model for predicting liver metastasis risk using differential methylation signals in primary CRC tumors, which can facilitate the decision for adjuvant chemotherapy.

**Methods:**

Fifty-nine stage I/II and IV CRC patients were enrolled. Primary tumor, adjacent normal tissue, and metastatic tumor tissues were subject to targeted bisulfite sequencing for DNA methylation. The Least Absolute Shrinkage and Selection Operator (LASSO) algorithm was used to identify potential DMRs for predicting liver metastasis of CRC.

**Results:**

We identified a total of 241,573 DMRs by comparing the DNA methylation profile of primary tumors of stage II patients who developed metastasis to those who were metastasis-free during the follow up period. 213 DMRs were associated with poor prognosis, among which 182 DMRS were found to be hypermethylated in the primary tumor of patients with metastases. Furthermore, by using the LASSO regression model, we identified 23 DMRs that contributed to a high probability of liver metastasis of CRC. The leave-one-out cross validation (LOOCV) was used to evaluate model predictive performance at an AUC of 0.701. In particular, 7 out of those 23 DMRs were found to be in the promoter region of genes that were previously reported prognostic biomarkers in diverse tumor types, including *TNNI2, PAX8, GUF1, KLF4, EVI2B, CEP112,* and long non-coding RNA *AC011298*. In addition, the model was also able to distinguish metastases of different sites (liver or lung) at an AUC of 0.933.

**Conclusion:**

We have identified DNA methylation biomarkers associated with the risk of cancer liver metastasis in early-stage CRC patients. A risk prediction model based on those epigenetic markers was proposed for outcome assessment.

**Supplementary Information:**

The online version contains supplementary material available at 10.1186/s13148-021-01108-3.

## Background

Colorectal cancer (CRC) is one of the most common malignancies worldwide and causes the fifth top cancer mortality in China [1, 2]. The incidence of CRC is associated with age-increasing and a slightly higher risk is found in men than women [3]. The 5-year survival rate for patients with localized CRC is optimistic, while it drops dramatically when distant metastases are developed [4]. Among all possible metastatic sites, liver is the most popular one due to the connection between the intestinal mesenteric drainage and the hepatic portal venous system [5]. Surgical resection is the only treatment needed for stage I CRC [4]. Considering the side effects and the potential benefits, adjuvant chemotherapy is not a recommended routine use in stage II CRC as the overall survival and disease-free survival were not significantly improved, but a decreased trend of tumor relapse was observed [6, 7]. Therefore, it is still debatable about the administration of adjuvant chemotherapies to postoperative stage II CRC patients. According to NCCN Guidelines, the risk assessment for adjuvant-treatment-decision is mainly based on clinical prognosis features such as poorly differentiated histology and lymphatic/vascular invasion. However, this may lead to overtreatment or miss the best timing of treatment as these features are insufficient for early prediction of metastasis. Identification of predictive biomarkers or high-risk molecular features to closely monitor disease progression and metastasis which are critical for determining whether the adjuvant therapy should be administrated is urgent for stage II CRC.

Next-generation sequencing is now playing an essential role in early disease detection and precision medicine. Besides genomic alterations, it has been applied to study the epigenetic changes during carcinogenesis and tumor metastasis. DNA methylation (DNAm) is one of the most investigated epigenetic mechanisms which establishes the genomic imprinting marks together with histone modifications and non-coding RNAs [8]. DNAm mainly occurs to the cytosine bases in the ‘CpG’ sites, which are enriched in gene promoters [9]. By pre-treating DNA with bisulfite, DNAm can be examined at a single-base resolution using high-throughput sequencing [10]. As aberrant DNAm patterns are considered to be associated with cancer and other diseases, researchers have put a lot of effort into identifying differentially methylated regions (DMRs) to investigate pathology and establish reliable biomarkers for prognosis prediction.

DMRs are widely studied as potential biomarkers for the diagnosis, prognosis, and treatment response in multiple cancer types. For instance, *OPCML* (opioid-binding cell adhesion molecule) and *FLRT2* (fibronectin leucine-rich transmembrane protein 2) were identified as novel DMRs to distinguish prostate tumors and normal tissues [11]. Similar results were found in lung cancer as well, where a list of DMR genes was identified in two patients by comparing tumor and normal tissue specimens [12]. The DNAm levels of a number of genes might contribute to the prediction of tumor progression in gastric cancer [13]. In ovarian cancer, hypermethylation of DMRs were detected in several tumor suppressor genes such as *ARHI* and *PEG3* [14]. While in CRC, a hypomethylated DMR in *IGF2* was reported to be associated with poor prognosis [15]*.* And a comprehensive methylation analysis of DMRs in CRC revealed the higher susceptibility of hypermethylation than hypomethylation in tumor tissues [16]. In this study, we sought to comprehensively study the DMR status in early-stage CRC patients and extracted dominant ones to establish a pilot model to predict liver metastases in stage II CRC.

## Methods and materials

### Patients recruitment and sample collection

We retrospectively studied CRC patients who received surgery on either primary or metastatic tumor at the Cancer Hospital, Chinese Academy of Medical Sciences between 2012 and 2018. An average 5-year follow-up evaluation was performed for each patient to confirm the status of liver metastasis till December 31, 2019. All patients have provided written informed consent. In total, 81 samples including tumor and tumor adjacent, were collected from 59 patients. All samples were shipped to the central laboratory of a clinical testing center (Nanjing Geneseeq Technology Inc., China) for targeted bisulfite sequencing.

### Targeted bisulfite sequencing and identification of differentially methylated region (DMR)

To construct the sequencing libraries, 1 μg of DNA per formalin-fixed paraffin-embedded (FFPE) sample was extracted and then treated with bisulfite. Targeted bisulfite sequencing was then performed on Illumina Hiseq platform (Illumina, San Diego, CA) using predesigned probes (SeqCap Epi CpGiant, Roche), which targeted a total of ~ 2.7 × 10^6^ CpG sites within ~ 80.5 Mb of genome region. Raw sequencing data were first demultiplexed by bck2fastq and then trimmed by Trimmomatic as part of the quality control (QC) protocol [17]. The qualified reads were then mapped onto the human reference genome (GRCh37/UCSC hg19) using the bisulfite sequence aligner Bismark [18] after PCR duplicates removal by Picard toolkit (http://broadinstitute.github.io/picard/).

The methylKit package (version 1.12.0) was used to identify DMRs in R (version 3.6.3) [19]. CpG clusters were tiled into 1000 bp windows with a minimum coverage ≥ 2 to ensure better detection of DMRs based on parameters which were previously reported by Ziller et al. [20]. The methylation level of each DMR was calculated using the total methylated cytosines divided by the total CpGs within each window. Logistic regression was applied to calculate the methylation difference as well as the false discovery rates (FDR) between the test and control groups.

### Identification of prognostic DMRs in stage II CRC patients

To identify potential methylation markers discriminating between stage II patients with favorable and unfavorable prognoses, we first identified DMRs using 38 stage II tumor samples (21 favorable vs 17 unfavorable). The DMRs, which were significantly different between samples with favorable and unfavorable prognoses (methylation differences ≥ 10%, q value ≤ 0.05), were selected for downstream analysis. We then annotated these DMRs using the latest gene annotation from the GENCODE project (release 19 for GRCh37/UCSC hg19) [21] and chose only DMRs located within the promoter regions, which were defined as 1500 bp upstream and 500 bp downstream of the transcription start sites, using BEDTools (version 2.26.0) [22]. Finally, we performed a Jonckheere trend test on the methylation levels of these DMRs, to identify any DMRs showing a significant trend in different cancer stages, including tumor-adjacent, stage I, II and IV. A total of 70 samples, which included the 38 stage II tumor samples as well as 32 additional samples (11 primary tumor adjacent, 11 stage I and 10 stage IV), were used and the final prognostic DMRs were selected if the trend was statistically significant (*p* value ≤ 0.05).

### Model predicting liver metastasis using primary CRC tumor samples

We used a total of 59 primary tumor samples to construct a model for predicting liver metastasis (LIM) in CRC patients. These 59 patient samples, as shown in Additional file 3: Table S1, included 22 patients with LIM and 37 patients without LIM during the follow-up. The machine learning approach was based on the stacked generalized linear model (GLM) of three based models using gradient boosting (GBM), Random Forest and Deep learning algorithms.

To increase the performance of our base model, we have performed tenfold cross-validation based on the training dataset to optimize the base models as well as the GLM stacked model. Each sample was used as a validation set for the LASSO model during the LOOCV, while the rest 58 samples were kept as the training set. The DMRs between primary tumor samples from LIM patients and LIM-free patients were identified using the training dataset only. A selective number of DMRs (methylation differences ≥ 20%, q value ≤ 0.05) were then used as candidates for identifying diagnostic DMRs. XGBoost was used to predict the performance of diagnostic DMRs, and the probability score for the validation set was calculated [23]. In total, the LASSO model was performed 59 times, and the Receiver Operating Characteristic (ROC) curve was constructed using probability scores of all 59 samples.

After the LOOCV was finished, the LASSO model was applied using the entire 59 primary tumor samples. A total of 105 DMRs between patients with/without LIM were identified using the aforementioned 59 primary tumor samples. After applying the LASSO algorithm, we were able to identify 23 DMRs from the total 105 candidate DMRs as the optimal diagnostic markers.

### Model evaluation using liver/lung metastasis tumor samples

To further evaluate the predicting power of our model, we used 6 liver metastasis tumor samples and 5 lung metastasis tumor samples from CRC patients. Among these 11 metastasis tumor samples, 21 of the 23 previously identified diagnostic markers were available. The probability scores of these 11 samples were calculated using the aforementioned predictive model. A ROC curve was constructed to evaluate the performance of the predictive model by examining if the liver metastasis status were correctly identified for the metastasis tumor samples.

## Results

### Clinical characteristics of the 59 CRC patients

In this study, a total of 59 patients who were diagnosed as CRC and treated at the Cancer Hospital, Chinese Academy of Medical Sciences were recruited. The general clinical characteristics of the cohort are summarized in Table 1. There were more male (36/59, 61.0%) than female (23/59, 39.0%) patients and the median age was 61 years old. The majority patients (42/59, 71.2%) were non-smokers. Nearly two-thirds (38/59, 64.4%) were initially diagnosed as stage II and 17 of them developed lung or liver metastasis (LIM:11, LUM: 6) during the follow-up period. Metastasis was also developed in two of the 11 stage I patients (LIM:1, LUM:1). All of the stage IV patients (N = 10) were initially diagnosed with LIM; meanwhile, LUM was found in two of them. To sum up, LIM were found in 22 of the 29 patients (75.9%) with metastatic tumors, making it more dominant than LUM (31.0%, 2 patients have both LIM and LUM). The median liver-metastasis-free survival time for these 59 patients was 2000 days, while the 1-year liver-metastasis-free rate was 81.4%, as shown in Additional file 1: Figure S1. A total of 81 tissue samples were collected from the entire cohort including 59 primary CRC tumor samples, 11 tumor-adjacent samples, and 11 metastatic tumor samples (6 LIM and 5 LUM) and the detailed information was summarized in Additional file 3: Table S1.

**Table 1:** Clinicopathological characteristics of the 59 CRC patients

Characteristic	Number (%)
**Overall**	59
**Median age (range)**	59 (40–78)
< 60	32 (54.2)
≥ 60	27 (45.8)
**Sex**	
Male	36 (61.0)
Female	23 (39.0)
**Smoke history**	
Smoker	16 (27.1)
Non-smoker	42 (71.2)
Unknown	1 (1.7)
**Stage**	
I	11 (18.7)
II	38 (64.4)
IV	10 (16.9)
**Metastasis**	
Stage I	
LIM	1 (9.1)
LUM	1 (9.1)
Stage II	
LIM	11 (28.9)
LUM	6 (15.8)
Stage IV	
LIM	10 (100) ^a^
LUM	2 (20) ^a^
All stages	
No metastasis	30 (50.8)

^a^Two patients have both liver and lung metastases

### Identify potential DMRs as prognostic markers

To identify any DMRs markers associated with prognosis, we sought to analyze the methylation status of primary tumor samples, which were grouped based on prognosis. To reduce the disturbance of stages, we only focused on the 38 stage II patients in which the number of favorable and unfavorable prognosis were similar.

**Fig. 1:**
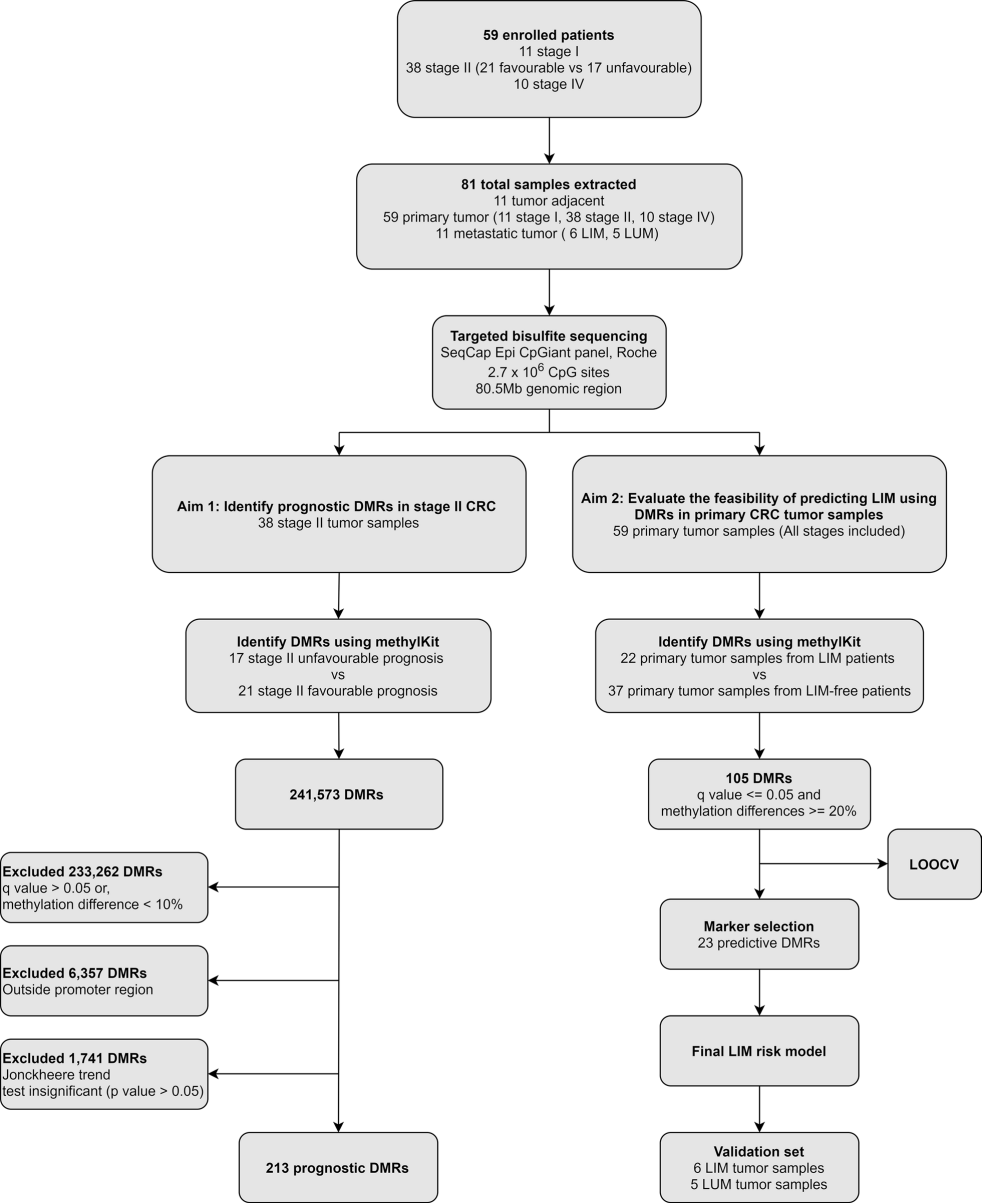
Flowchart for identifying prognostic and predictive DMRs. Targeted bisulfite sequencing was performed on a total of 81 samples containing 59 primary CRC tumor samples (11 stage I, 38 stage II, 10 stage IV), as well as 11 tumor-adjacent samples and 11 metastatic tumor samples. Aim 1. prognostic marker selection: DMRs were identified between the stage II unfavorable group (17 samples) and the stage II favorable prognosis group (21 samples). Jonckheere trend test was performed on 70 samples including 11 tumor adjacent and 59 primary samples. 213 DMRs were selected as the final prognostic markers. Aim 2: LIM predictive marker selection: a LASSO-based LOOCV was applied to a training cohort of 59 primary CRC tumor samples to identify a final selection of 23 DMR markers. These 23 markers were then validated by a dataset of 6 LIM and 5 LUM tumor samples. LIM: liver metastasis; LUM: lung metastasis

These 38 stage II patients were grouped into a test and control group to identify DMRs using the methylkit package (version 1.12.0) in R (version 3.6.3) [19]. The test group was comprised of 17 patients with unfavorable prognosis, who developed either LIM or LUM during the follow-up period. The other 21 patients without metastases were grouped into the control group. The primary tumor samples of these 38 stage II CRC patients were used to identify DMRs as described in Fig. 1. We were able to identify a total of 241,573 DMRs (detail data not shown) between the test group and the control group.

**Fig. 2:**
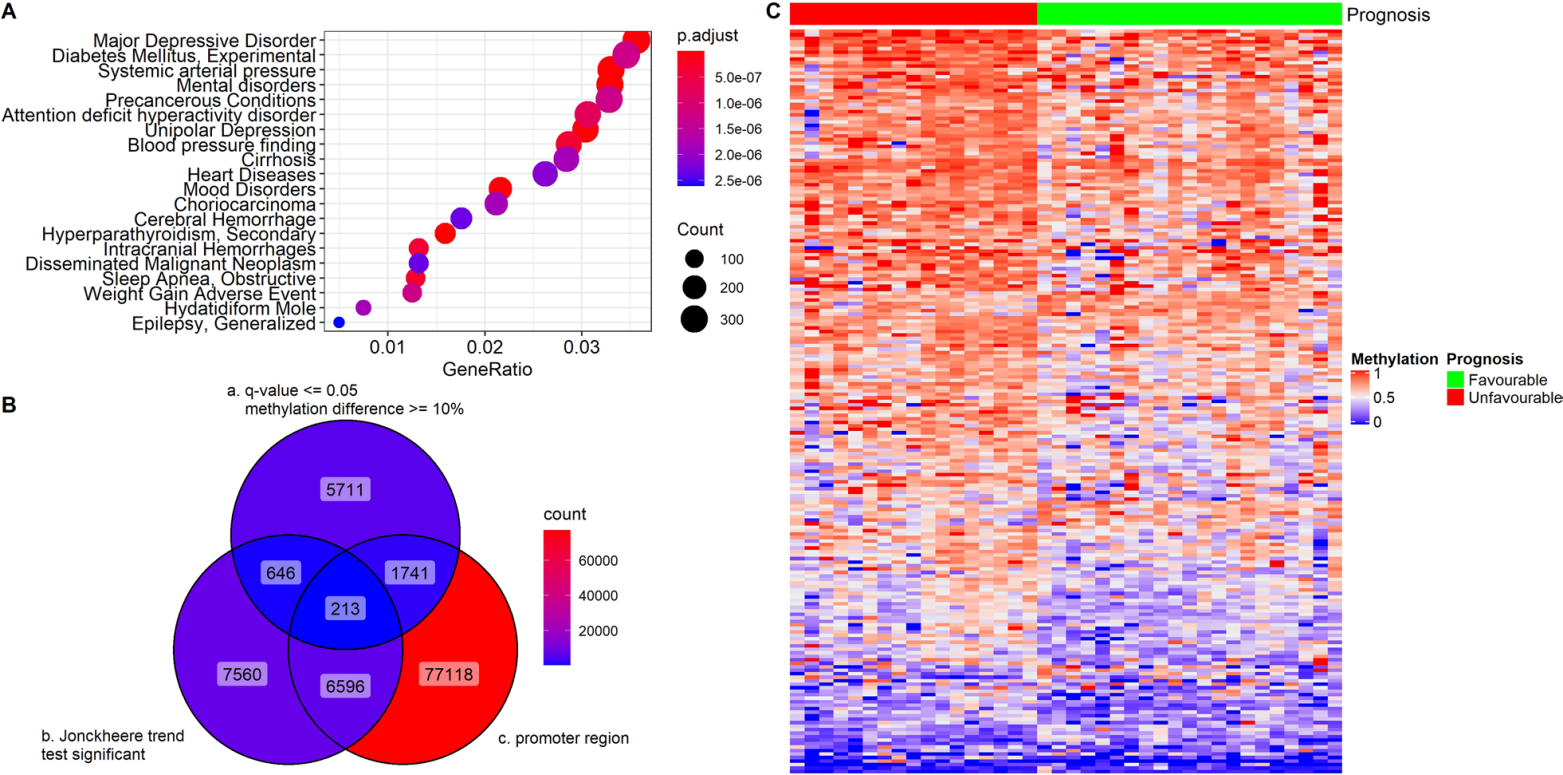
DMR analysis of Stage II CRC prognosis. **A** Dotplot of enriched terms using DMRs among Stage II favorable prognosis and Stage II unfavorable prognosis tumor samples. A total of 19,809 genes which had DMR in the promoter regions were used for the enrichment analysis of disease-gene associations. **B** Venn diagram for selecting the final 213 prognostic DMR markers. a) 8,311 candidate DMRs between Stage II favorable and unfavorable prognosis samples (q value ≤ 0.05 & methylation differences ≥ 10%); b); 15,015 DMRs shown statistic significances (*p* value ≤ 0.05) in Jonckheere trend test among 59 CRC tumor and 11 tumor -adjacent samples; c) 85,668 DMRs overlapping with a gene promoter region (1500 bp upstream/500 downstream of transcription start site); **C** Heatmap of the final 213 prognosis marker methylation values in 21 Stage II favorable and 17 unfavorable prognosis tumor samples

To better understand their potential functional impact, we then annotated our identified DMRs against the latest gene annotation for GRCh37/UCSC hg19 from the GENCODE project (release 19) [21]. In total, 85,668 DMRs were found to be overlapping with a promoter region (Fig. 2B), which was defined as 1500 bp upstream to 500 bp downstream of a transcription start site. We then performed a pathway enrichment test using a subset of 26,105 DMRs, of which the q values met the cut-off of 0.05 (Fig. 2A). These 26,105 DMRs overlapped with the promoter regions of 19,809 genes, which were then used as the input for the enrichment analysis of disease-gene associations in the R package clusterProfiler [24]. The top 20 enriched disease terms are shown in Fig. 2A. These enriched terms included precancerous conditions and disseminated malignant neoplasm, which could be linked to the development of metastatic tumors. It was worth noting that cirrhosis, which was evidently diagnosed within patients with liver tumors [25], was also found among the enriched terms in our results. This was possibly contributed by the fact that the majority (64.7%, 11/17) of the unfavorable prognosis patients were diagnosed with LIM instead of LUM (Additional file 3: Table S1).

To further validate the prognostic power of these DMRs, we then performed the Jonckheere trend test using methylation value in the 59 primary tumor samples of different cancer stages as well as in the 11 tumor-adjacent samples from the 59 CRC patients. For the trend test, these 70 samples were clustered into 4 groups based on their TNM stage information (tumor adjacent, stage I, II and IV, respectively). We used 241,199 DMRs from the total 241,573 DMRs, which had methylation values available in all 70 samples (detail data not shown). For each DMR, a Jonckheere trend test was performed and 15,015 DMRs which were showing statistical significance were selected for downstream analysis (Fig. 2B). We then selected 8,311 candidate DMRs, which were showing significant differences (q value ≤ 0.05, methylation differences ≥ 10%) between methylation values of the test group and the control group, from the total 241,573 DMRs for further analysis (Fig. 2B).

A final set of 213 DMRs (Additional file 3: Table S2), which met all three of the aforementioned filtering criteria, were selected as the prognostic DMR markers, as shown by the Venn diagram in Fig. 2. We then constructed a heatmap using the methylation values of these 213 DMRs in the 38 stage II primary tumor samples. As shown in Fig. 2C, there was noticeable differences between the group of unfavorable prognosis and favorable prognosis. These 213 DMRs between the 17 unfavorable prognosis and 21 favorable prognosis samples, as shown in Additional file 3: Table S2, included 182 hypermethylated and 31 hypomethylated DMRs. The overwhelming number of hypermethylated DMRs in our results was consistent with the current understanding that DNA hypermethylation associated with cancer are mostly found in gene regions, despite there are more hypomethylation compared to hypermethylation in general [26]. The targeted bisulfite sequencing was performed using SeqCap Epi CpGiant probes, which is biased toward finding hypermethylation as it focused more on genic regions compared to the whole genome bisulfite sequencing approach.

**Fig. 3:**
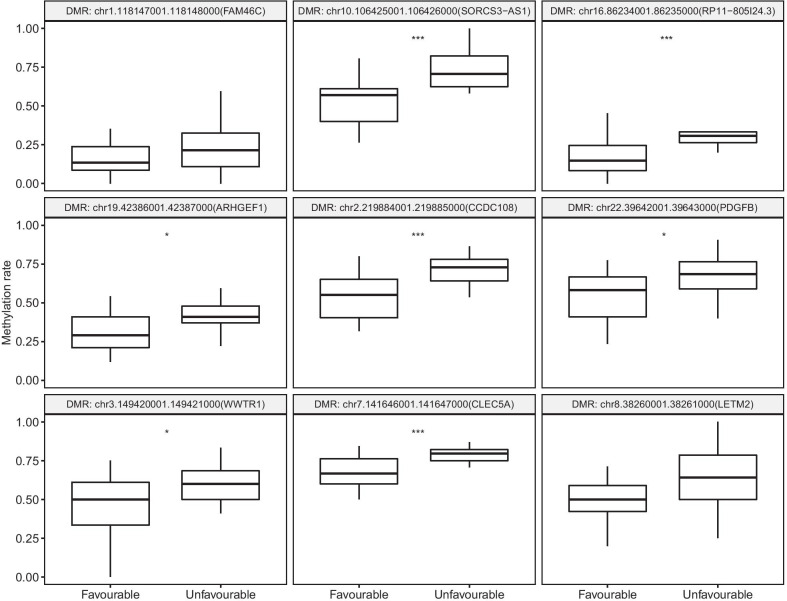
Methylation values of nine DMRs in Stage II favorable and unfavorable prognosis primary tumor samples. Data were analyzed using student t test. *p < 0.05, ***p < 0.001

Additionally, the 213 prognostic DMRs have been filtered against gene region and are overlapping with promoter regions, which can enhance such bias. Figure 3 shows the comparison of methylation rate of nine randomly chosen prognostic DMRs between favorable and unfavorable prognosis groups. All of them were susceptible to be hypermethylated when metastasis was developed later on and mostly located in promoter regions of genes which had confirmed associations with certain type of cancer.

We then performed statistical analysis to investigate the potential impact of age and smoking history had on the 213 identified prognostic DMRs. The 38 stage II patients were first split into two different groups comparing their age toward the group median (< 60 years old and ≥ 60 years old, 16 and 22 patients, respectively). For each of the 213 DMRs, raw methylation rates for the 38 stage II patients were used for wilcoxon test to compare if the two groups are significantly different (fdr adjusted *p* value or *q* value < 0.1). None of the 213 DMRs showed any statistical significance differences between the two groups, as shown in Additional file 3: Table S3. Similar to the age test, these patients, excluding one patient without smoking history information, were subsequently grouped into smoking [11] and non-smoking [26] groups. The Wilcoxon tests shown that none of these DMRs had significant differences based on patients smoking history (*q* value < 0.1, Additional file 3: Table S3).

### Construct a pilot model for predicting the liver metastasis based on DMRs in primary colorectal tumor

**Fig. 4:**
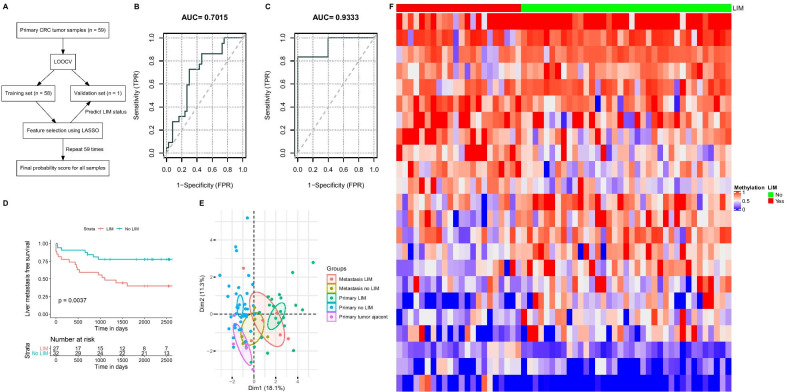
DMR analysis of predicting future LIM status using CRC primary tumors. **A** Flow chart for predictive DMRs using LASSO-based LOOCV. **B** Receiver operating characteristic curve of the 23 predictive DMR markers in 59 primary CRC tumor samples. **C** Receiver operating characteristic curve of the 23 predictive DMR markers in 11 metastasis CRC tumor samples (6 LIM, 5 LUM). **D** Liver metastasis-free survival for the 59 CRC patients with and without LIM. **E** Principal Component Analysis of the 23 predictive DMRs in 81 samples. **F** Heatmap of the 23 predictive DMR marker methylation values in 59 primary tumor samples with future LIM status

Since LIM is the more dominant type of metastasis among CRC patients, we set to explore the possibility to predict LIM status using the primary tumor samples. The total 59 primary tumor samples were used to construct a pilot model for predicting the LIM status in these CRC patients. A total of 105 DMRs (methylation differences ≥ 20%, q value ≤ 0.05) were identified between the 22 patients with LIM and 37 patients without LIM during the follow-up. These DMRs were then used as candidates for the predictive model. By utilizing LOOCV and LASSO model as described in Fig. 4A, we were able to generate the probability scores for all 59 samples. A ROC curve was constructed, as shown in Fig. 4B, using these probability scores, yielding an Area Under Curve (AUC) score of 0.7015 (sensitivity = 72.7%, specificity = 70.3%, as shown in Additional file 3: Table S4). The model was then applied to the total 59 primary samples, and 23 out of the 105 DMRs were identified as optimal predictive markers (shown in Table 2). 7 of 23 (30.4%) were overlapped with a promoter region and 6 (26.1%) were located within the coding region. The rest 7 DMRs were involved in the intergenic regions. Wilcoxon test results shown that none of these 23 DMRs were significantly different between different age/smoking group within these patients (*q* value < 0.1, Additional file 3: Table S5).

**Table 2:** Annotation of the 23 predictive DMRs identified using 59 primary CRC tumor samples

Genome position	Gene context	Gene name	Type of DMR
chr11:1861001–1862000	Promoter	TNNI2	hypo
chr2:113994001–113995000	Promoter	PAX8	hypo
chr2:241626001–241627000	Promoter	AC011298	hypo
chr4:44679001–44680000	Promoter	GUF1	hyper
chr9:110248001–110249000	Promoter	KLF4	hyper
chr17:29641001–29642000	Promoter	EVI2B	hyper
chr17:63739001–63740000	Promoter	CEP112	hypo
chr1:200977001–200978000	Gene body	KIF21B	hypo
chr12:66765001–66766000	Gene body	GRIP1	hyper
chr13:111289001–111290000	Gene body	NAXD	hypo
chr19:39314001–39315000	Gene body	ECH1	hypo
chr2:136279001–136280000	Gene body	ZRANB3	hyper
chr7:51148001–51149000	Gene body	COBL	hypo
chr8:72468001–72469000	Gene body	RP11-1102P16.1	hyper
chr8:99394001–99395000	Gene body	KB-1458E12.1	hypo
chr16:1344001–1345000	Gene body	RP11-616M22.7	hypo
chr11:12097001–12098000	Intergenic	N/A	hyper
chr12:90907001–90908000	Intergenic	N/A	hypo
chr2:75504001–75505000	Intergenic	N/A	hypo
chr22:18530001–18531000	Intergenic	N/A	hyper
chr4:153039001–153040000	Intergenic	N/A	hypo
chr7:34344001–34345000	Intergenic	N/A	hyper
chr9:139591001–139592000	Intergenic	N/A	hypo

To explore the utility of the established model in discriminating metastatic sites, we generated another ROC curve using the 23 predictive markers on 11 metastatic tumor samples (6 LIM and 5 LUM). The model was showing an excellent performance in discriminating the LIM samples against the LUM samples (AUC = 0.9333, sensitivity = 83.3%, specificity = 100%, Fig. 4C). Furthermore, a Kaplan–Meier curve of LIM-free survival suggested that the patient predicted as LIM positive had a significantly shorter LIM-free survival compared to the LIM predicted negative (Log-rank *p* = 0.0037, Fig. 4D). We also constructed a Principal Component Analysis (PCA) with the total 81 samples including 11 tumor-adjacent samples, 59 primary tumor samples and 11 metastatic tumor samples. As illustrated by the PCA (Fig. 4E), the LIM positive and LIM negative groups in the primary tumor samples were separated from each other. Similarly, the LIM positive and negative samples from the metastatic tumor can be distinguished. The 11 tumor-adjacent samples were placed closer to the primary without LIM samples yet with trend of separation. Finally, visible differences can be observed in the heatmap generated using these 23 DMRs in the 59 primary tumor samples (Fig. 4F). Finally, an external validation cohort, which consist of primary tumor samples from 8 CRC patients (4 LIM and 4 LIM-free), was used to further evaluate the model performance. As shown in Additional file 2: Figure S2, our model showed great performance in differentiating the LIM samples from the LIM-free samples (AUC = 0.875, sensitivity = 100%, specificity = 75.0%).

## Discussion

Despite detail mechanisms not being completely understood, DNAm are believed to have the ability of altering downstream gene expression by affecting the binding of transcription factors and their target sites[27]. Additionally, evidence suggest that DNAm within the gene body, especially the first exon, can be associated with transcriptional silencing[28]. DNAm now is an emerging biomarker for cancer diagnosis and prognosis prediction which plays an important role in establishing epigenetic imprints.

Here we investigated the different DNAm status in CRC patients with and without metastasis within the follow-up period. Using targeted bisulfite sequencing, we initially identified over 24 thousand of DMRs by comparing the primary tumor samples of 38 stage II patients. With several steps of data filtration, we eventually selected 213 candidate DMRs which might serve as metastasis predictors and the majority were hypermethylated in the unfavorable prognosis group. Evidence suggested that, while there are more cancer-related hypomethylation than hypermethylation in the intergenic region, DNAm associated with cancer found in genic regions were mostly hypermethylation [26, 29]. A recent study in 2018 by Hidaka et. al has focused on genes expression regulated by DMRs in 106 CRC patients and found an overall trend toward hypermethylation in CRC tissue samples [16]. This agrees with the fact that we had more hypermethylation in our results, which were all located within promoter regions further enhancing such bias.

Through literature searching, we found that many of them were associated with cancer-related genes. For example, our results suggested that the unfavorable prognosis group were hypermethylated compared to the favorable prognosis group within the region chr1:118147001–118148000. Such hypermethylation in the promoter region would in theory result in down-regulation of the *FAM46C* gene, which was acting as an onco-suppressor gene in multiple myeloma [30]. Similarly, our data suggested that the promoter regions of *SORCS3 − AS1* were differentially methylated among the two groups of different prognoses. Interestingly, Schneider et al. reported in 2015 that the methylation level of *SORCS3* gene can be associated with tumor progression in gastric cancer [13]. The promoter region of *HLA-DQA1* gene, which was part of the human leukocyte antigen (*HLA*) complex, was differentially methylated and labeled as a prognostic marker in our result. Intriguingly, the expression of *HLAB* gene, which was also part of the *HLA* complex, was reportedly associated with tumor progression in CRC [31]. Furthermore, *ARHGEF1*, *CFAP65*, *PDGFRB* and *CLEC5A* were labeled as prognostic marker by the Human Protein Atlas in renal and breast cancer, endometrial cancer, renal and urothelial cancer, and ovarian cancer, respectively [32, 33].

The predictive model established through LASSO and LOOCV was based on 23 DMRs, most of them were located in the gene coding or promoter regions. The seven genes whose promoter contained these DMRs were either previously reported as prognostic markers or showed the predictive potential in multiple cancers. For example, *PAX8* and *GUF1* were reported as prognostic markers for endometrial cancer, renal cancer and thyroid cancer, respectively [33]. Both *KLF4* and *EVI2B* were identified by the Human Protein Atlas as markers for renal cancer prognoses (favorable and unfavorable, respectively) [33]. Furthermore, *TNNI2* was reported to have predictive power for metastatic tumor development in gastric cancer [34]. A recent study suggested that the centrosomal protein 112 (*Cep112*) was able to act as an oncogene by interacting with genomic instability inducing RNA [35]. Finally, the long non-coding RNA *AC011298* was among the six identified prognostic markers identified in a bladder cancer study [36].

In clinical setting, the postoperative treatment-decision for early-stage CRC is challenging. The high odd of metastasis development especially to liver dramatically decreases the five-year survival rate. Therefore, early-prediction of liver metastasis could be powerful to improve prognosis for early-stage CRC patients. Many biomarkers have been investigated for the possibility of predicting metastasis such as microRNAs [37] and specific gene expression level [38]. Nowadays, epigenetic information has drawn a broad attention as predictive biomarkers and DNAm status is the most investigated. Previous studies have identified hundreds of DMRs by comparing different conditions such as stage, prognosis, and histology. However, no solid predictive model has been established. Herein, we sought to explore the possibility of predicting liver metastasis based on the primer tumor DNAm profiles. Taking advantages of the surgery resected tissue biopsy to predict the possibility of metastasis could reduce overtreatment and provide valuable information to guide treatment. We explored the DNAm profiles of the primary tumors to characterize novel DMRs features by comparing favorable and unfavorable stage II CRC patients. By identifying potential DMR markers which could reflect the risk of liver metastasis, we aimed to eventually establish a model to predict the metastasis risk by detecting the primary tumor DMRs. However, due to the restricted cohort size, we could only perform the LOOCV for model selection and validated the predictive model based on the metastatic tumor samples and a small external validation cohort. A larger cohort with methylation values in primary CRC samples would be a great value for DMRs identification and modelling which remained to be completed in the future. Therefore, in the present study, we have identified DNAm biomarkers associated with the risk of cancer liver metastasis in early-stage CRC patients and proposed a pilot risk prediction model based on those epigenetic markers for outcome assessment.

## Supplementary Information

**Additional file 1: Figure S1**. Liver metastasis free survival for the 59 CRC patients. 60% of the 59 CRC patients reached a stable stage of liver metastasis free at 1600 days.

**Additional file 2: Figure S2**. Receiver operating characteristic curve in an external validation cohort. Performance of the 23 predictive DMR markers were tested using 8 primary CRC tumor samples (4 LIM, 4 LIM-free).

**Additional file 3: Table S1**. Clinical characteristics of the 81 samples from 59 CRC patients included in this study. **Table S2**. Final 213 prognositc DMRs in Stage II CRC patients. **Table S3**. Wilcox test results between different age/smoking group for the 213 prognositic DMRs. **Table S4**. LOOCV model performances. **Table S5**. Wilcox test results between different age/smoking group for the 23 LIM-predictive DMRs.

## Data Availability

The datasets used and/or analyzed during the current study are available from the corresponding author on reasonable request.

## References

[1] Siegel RL, Miller KD, Goding Sauer A, Fedewa SA, Butterly LF, Anderson JC (2020). Colorectal cancer statistics, 2020. CA Cancer J Clin.

[2] Chen W, Zheng R, Baade PD, Zhang S, Zeng H, Bray F (2016). Cancer statistics in China, 2015. CA Cancer J Clin.

[3] Wong MCS, Huang J, Lok V, Wang J, Fung F, Ding H, et al. Differences in incidence and mortality trends of colorectal cancer worldwide based on sex, age, and anatomic location. Clin Gastroenterol Hepatol. 2020. 10.1016/j.cgh.2020.02.026 32088300

[4] Siegel RL, Miller KD, Jemal A (2020). Cancer statistics, 2020. CA A Cancer J Clin.

[5] Zarour LR, Anand S, Billingsley KG, Bisson WH, Cercek A, Clarke MF (2017). Colorectal cancer liver metastasis: evolving paradigms and future directions. Cell Mol Gastroenterol Hepatol.

[6] Schippinger W, Samonigg H, Schaberl-Moser R, Greil R, Thodtmann R, Tschmelitsch J (2007). A prospective randomised phase III trial of adjuvant chemotherapy with 5-fluorouracil and leucovorin in patients with stage II colon cancer. Br J Cancer.

[7] Jalaeikhoo H, Zokaasadi M, Khajeh-Mehrizi A, Rajaeinejad M, Mousavi SA, Vaezi M (2019). Effectiveness of adjuvant chemotherapy in patients with Stage II colorectal cancer: a multicenter retrospective study. J Res Med Sci.

[8] Ferguson-Smith AC (2011). Genomic imprinting: the emergence of an epigenetic paradigm. Nat Rev Genet.

[9] Illingworth RS, Bird AP (2009). CpG islands–'a rough guide'. FEBS Lett.

[10] Mallik S, Odom GJ, Gao Z, Gomez L, Chen X, Wang L (2019). An evaluation of supervised methods for identifying differentially methylated regions in Illumina methylation arrays. Brief Bioinform.

[11] Wu Y, Davison J, Qu X, Morrissey C, Storer B, Brown L (2016). Methylation profiling identified novel differentially methylated markers including OPCML and FLRT2 in prostate cancer. Epigenetics.

[12] Hong Y, Hong S-H, Oh Y-M, Shin S-H, Choi SS, Kim WJ (2018). Identification of lung cancer specific differentially methylated regions using genome-wide DNA methylation study. Mol Cell Toxicol.

[13] Schneider BG, Mera R, Piazuelo MB, Bravo JC, Zabaleta J, Delgado AG (2015). DNA methylation predicts progression of human gastric lesions. Cancer Epidemiol Biomark Prev.

[14] Feng W, Marquez RT, Lu Z, Liu J, Lu KH, Issa JP (2008). Imprinted tumor suppressor genes ARHI and PEG3 are the most frequently down-regulated in human ovarian cancers by loss of heterozygosity and promoter methylation. Cancer.

[15] Baba Y, Nosho K, Shima K, Huttenhower C, Tanaka N, Hazra A (2010). Hypomethylation of the IGF2 DMR in colorectal tumors, detected by bisulfite pyrosequencing, is associated with poor prognosis. Gastroenterology.

[16] Hidaka H, Higashimoto K, Aoki S, Mishima H, Hayashida C, Maeda T (2018). Comprehensive methylation analysis of imprinting-associated differentially methylated regions in colorectal cancer. Clin Epigenet.

[17] Bolger AM, Lohse M, Usadel B (2014). Trimmomatic: a flexible trimmer for Illumina sequence data. Bioinformatics.

[18] Krueger F, Andrews SR (2011). Bismark: a flexible aligner and methylation caller for Bisulfite-Seq applications. Bioinformatics.

[19] Akalin A, Kormaksson M, Li S, Garrett-Bakelman FE, Figueroa ME, Melnick A (2012). methylKit: a comprehensive R package for the analysis of genome-wide DNA methylation profiles. Genome Biol.

[20] Ziller MJ, Hansen KD, Meissner A, Aryee MJ. Coverage recommendations for methylation analysis by whole-genome bisulfite sequencing. Nat Methods. 2015;12(3):230–2, 1 p following 2. 10.1038/nmeth.3152 PMC4344394 25362363

[21] Harrow J, Frankish A, Gonzalez JM, Tapanari E, Diekhans M, Kokocinski F (2012). GENCODE: the reference human genome annotation for The ENCODE Project. Genome Res.

[22] Quinlan AR, Hall IM (2010). BEDTools: a flexible suite of utilities for comparing genomic features. Bioinformatics.

[23] Chen T, Guestrin C. XGBoost: a scalable tree boosting system. Proceedings of the 22nd ACM SIGKDD international conference on knowledge discovery and data mining; San Francisco, California, USA: Association for Computing Machinery; 2016. p. 785–94.

[24] Yu G, Wang LG, Han Y, He QY (2012). clusterProfiler: an R package for comparing biological themes among gene clusters. OMICS.

[25] Kanda T, Goto T, Hirotsu Y, Moriyama M, Omata M. Molecular mechanisms driving progression of liver cirrhosis towards hepatocellular carcinoma in chronic hepatitis B and C infections: a review. Int J Mol Sci. 2019;20(6). 10.3390/ijms20061358 PMC6470669 30889843

[26] Ehrlich M (2002). DNA methylation in cancer: too much, but also too little. Oncogene.

[27] Kerachian MA, Javadmanesh A, Azghandi M, Mojtabanezhad Shariatpanahi A, Yassi M, Shams Davodly E (2020). Crosstalk between DNA methylation and gene expression in colorectal cancer, a potential plasma biomarker for tracing this tumor. Sci Rep.

[28] Brenet F, Moh M, Funk P, Feierstein E, Viale AJ, Socci ND, et al. DNA methylation of the first exon is tightly linked to transcriptional silencing. PLoS ONE. 2011;6(1):e14524. 10.1371/journal.pone.0014524 PMC3022582 21267076

[29] Frigola J, Solé X, Paz MF, Moreno V, Esteller M, Capellà G (2005). Differential DNA hypermethylation and hypomethylation signatures in colorectal cancer. Hum Mol Genet.

[30] Mroczek S, Chlebowska J, Kuliński TM, Gewartowska O, Gruchota J, Cysewski D (2017). The non-canonical poly(A) polymerase FAM46C acts as an onco-suppressor in multiple myeloma. Nat Commun.

[31] Kirana C, Peng L, Miller R, Keating JP, Glenn C, Shi H (2019). Combination of laser microdissection, 2D-DIGE and MALDI-TOF MS to identify protein biomarkers to predict colorectal cancer spread. Clin Proteom.

[32] Ma Y, Chen X, Wang A, Zhao H, Lin Q, Bao H, et al. Copy number loss in granzyme genes confers resistance to immune checkpoint inhibitor in nasopharyngeal carcinoma. J Immunother Cancer. 2021;9(3). 10.1136/jitc-2020-002014 PMC7978327 33737344

[33] Uhlén M, Fagerberg L, Hallström BM, Lindskog C, Oksvold P, Mardinoglu A (2015). Proteomics. Tissue-based map of the human proteome. Science.

[34] Sawaki K, Kanda M, Miwa T, Umeda S, Tanaka H, Tanaka C (2018). Troponin I2 as a Specific Biomarker for Prediction of Peritoneal Metastasis in Gastric Cancer. Ann Surg Oncol.

[35] Panda S, Setia M, Kaur N, Shepal V, Arora V, Singh DK, et al. Noncoding RNA Ginir functions as an oncogene by associating with centrosomal proteins. PLoS Biol. 2018;16(10):e2004204. 10.1371/journal.pbio.2004204 PMC6193740 30296263

[36] Gao X, Zhang S, Chen Y, Wen X, Chen M, Wang S (2019). Development of a novel six-long noncoding RNA signature predicting survival of patients with bladder urothelial carcinoma. J Cell Biochem.

[37] Hur K, Toiyama Y, Okugawa Y, Ide S, Imaoka H, Boland CR (2017). Circulating microRNA-203 predicts prognosis and metastasis in human colorectal cancer. Gut.

[38] Wang X, Liu X, Li AY, Chen L, Lai L, Lin HH (2011). Overexpression of HMGA2 promotes metastasis and impacts survival of colorectal cancers. Clin Cancer Res.

