# Supplementary figures and images for: Identification of DNA methylation biomarkers for risk of liver metastasis in early-stage colorectal cancer
Source: Clin Epigenetics. 2021 Jun 9;13:126. doi: 10.1186/s13148-021-01108-3 (PMC8190869; doi:10.1186/s13148-021-01108-3)

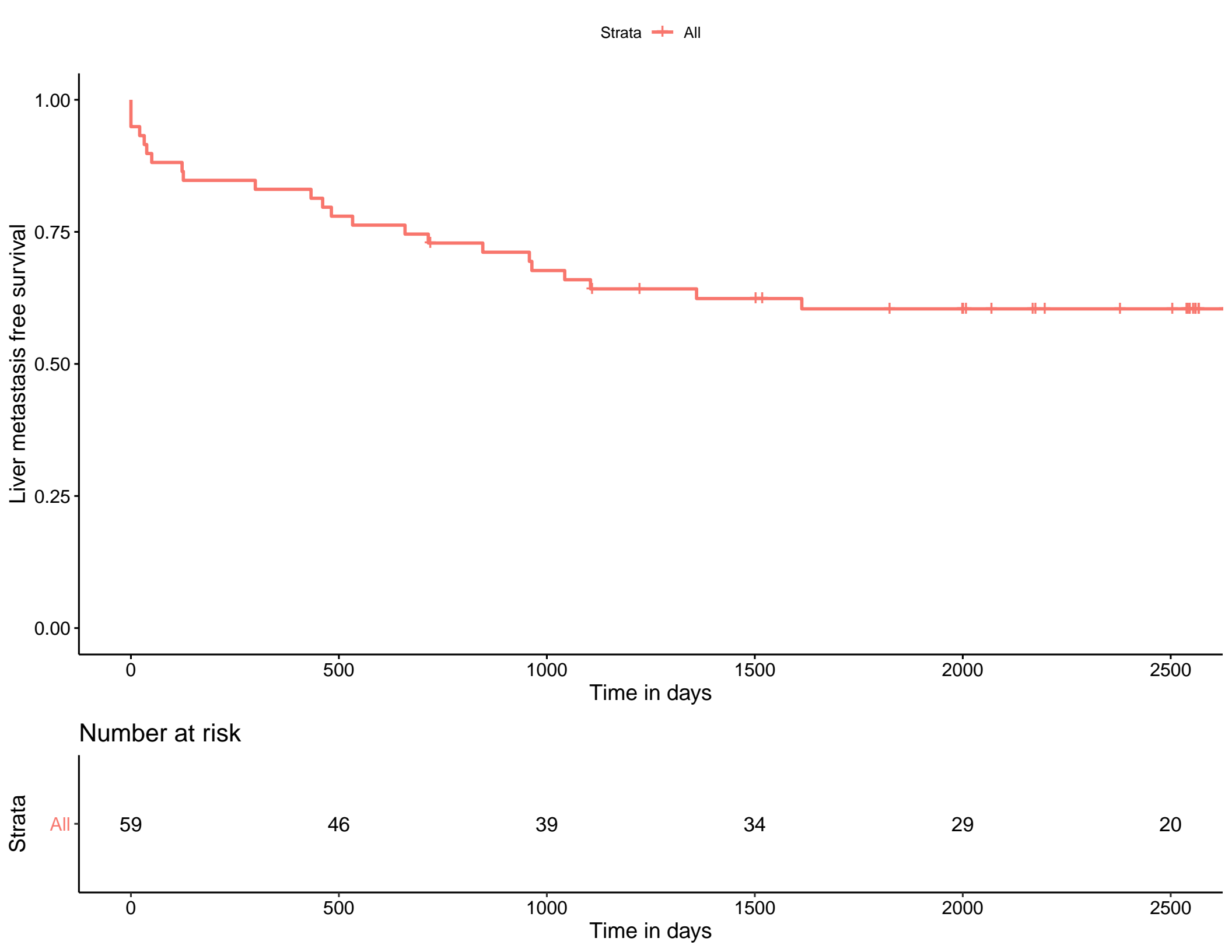

Supplement: Supplementary file 1 — Additional file 1: Figure S1. Liver metastasis free survival for the 59 CRC patients. 60% of the 59 CRC patients reached a stable stage of liver metastasis free at 1600 days. [file 13148_2021_1108_MOESM1_ESM.pdf]

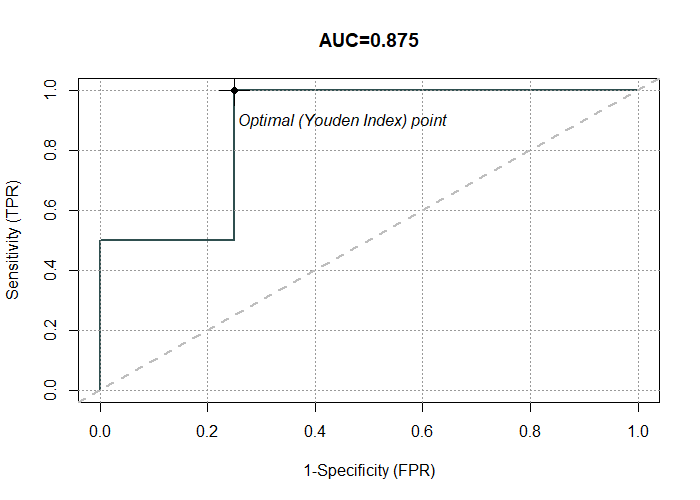

Supplement: Supplementary file 2 — Additional file 2: Figure S2. Receiver operating characteristic curve in an external validation cohort. Performance of the 23 predictive DMR markers were tested using 8 primary CRC tumor samples (4 LIM, 4 LIM-free). [file 13148_2021_1108_MOESM2_ESM.png]
